# Molecular evolution of pentatricopeptide repeat genes reveals truncation in species lacking an editing target and structural domains under distinct selective pressures

**DOI:** 10.1186/1471-2148-12-66

**Published:** 2012-05-14

**Authors:** Michael L Hayes, Karolyn Giang, R Michael Mulligan

**Affiliations:** 1 Developmental & Cell Biology, University of California, Irvine, USA; 2 University of California, 5217 McGaugh Hall, Irvine, CA 92697, USA

**Keywords:** PPR, Truncation, RNA editing, Brassicaceae, dN/dS, Alpha helix

## Abstract

**Background:**

Pentatricopeptide repeat (PPR) proteins are required for numerous RNA processing events in plant organelles including C-to-U editing, splicing, stabilization, and cleavage. Fifteen PPR proteins are known to be required for RNA editing at 21 sites in Arabidopsis chloroplasts, and belong to the PLS class of PPR proteins. In this study, we investigate the co-evolution of four PPR genes (*CRR4*, *CRR21*, *CLB19*, and *OTP82*) and their six editing targets in Brassicaceae species. PPR genes are composed of approximately 10 to 20 tandem repeats and each repeat has two α-helical regions, helix A and helix B, that are separated by short coil regions. Each repeat and structural feature was examined to determine the selective pressures on these regions.

**Results:**

All of the PPR genes examined are under strong negative selection. Multiple independent losses of editing site targets are observed for both *CRR21* and *OTP82*. In several species lacking the known editing target for *CRR21,* PPR genes are truncated near the 17th PPR repeat. The coding sequences of the truncated *CRR21* genes are maintained under strong negative selection; however, the 3’ UTR sequences beyond the truncation site have substantially diverged. Phylogenetic analyses of four PPR genes show that sequences corresponding to helix A are high compared to helix B sequences. Differential evolutionary selection of helix A versus helix B is observed in both plant and mammalian PPR genes.

**Conclusion:**

PPR genes and their cognate editing sites are mutually constrained in evolution. Editing sites are frequently lost by replacement of an edited C with a genomic T. After the loss of an editing site, the PPR genes are observed with three outcomes: first, few changes are detected in some cases; second, the PPR gene is present as a pseudogene; and third, the PPR gene is present but truncated in the C-terminal region. The retention of truncated forms of CRR21 that are maintained under strong negative selection even in the absence of an editing site target suggests that unrecognized function(s) might exist for this PPR protein. PPR gene sequences that encode helix A are under strong selection, and could be involved in RNA substrate recognition.

## Background

Pentatrico peptide repeat (PPR) proteins are present in yeasts, mammals, plants, protists and are thought to be present in most eukaryotes [1,2]. All of these organisms have P class PPR proteins that are characterized by tandem repeats of a degenerate 35 amino acid motif. The PPR family of proteins has greatly expanded in land plants [1]. In addition to the P class of PPR proteins, roughly half of land plant PPR proteins have a repeating tripartite sequence of P, L, and S type PPR repeats [1]. PLS type proteins typically have additional C-terminal domains known as the E, E + and DYW domains [1]. The DYW domain may be the catalytic deaminase domain based on the conserved residues that are similar to cytidine deaminases, evolutionary co-incidence with RNA editing, and structural models that suggest a deaminase-like conformation [3-5]. PPR proteins are known to be required for RNA editing, splicing, stabilization, and cleavage in plant organelles [6-11].

Cytidines are edited to uridines in many mitochondrial and chloroplasts transcripts [12,13]. RNA editing typically leads to the maintenance of an evolutionary conserved codon at the RNA level, and is thought to repair errors that were allowed to accumulate in the genome [14]. In the plant *A. thaliana,* fifteen PPR proteins have been identified that are necessary for editing 21 of the 34 known chloroplast RNA editing sites [8,15-21]. The genes that have been shown to be required for chloroplast editing are PLS type PPR proteins and include at least the C-terminal E domain, while the presence of the E + and DYW domains varies. Disruption of some PPR genes that are required for editing results in photosynthetic defects [8,15,16,19,21], while disruption of other PPR genes lead to weak phenotypes in spite of the elimination of editing at one or more editing sites [17,18]. Although detailed mechanisms for RNA binding are not known, amino acid changes in PPR proteins have been associated with changes in editing efficiency [22-25], and this observation suggests that there is significant co-evolution between nuclear-encoded editing factors and organellar RNA *cis*-elements. Editing activity for an exogenous site was observed in a plant that lacked an endogenous target, suggesting that the conservation of the editing activity occurred independently from an editing target [26].

Loss of cognate editing sites for a PPR has been shown to result in pseudogene formation that was apparently the result of the loss of evolutionary selection in the absence of the target editing site [27]. In this study, we show that multiple independent losses of an editing site target correlates with truncation of a PLS type PPR gene and elimination of the editing-associated E/E + domains in the C-terminus. Unexpectedly, even in the absence of the known editing site target, the truncated PPR genes are maintained under strong negative selection. The maintenance of strong negative selection suggests that these orphan PPR genes might have an additional unrecognized function that is independent of the known chloroplast RNA editing function.

## Results

### **The evolution of editing sites associated with four Brassicaceae PPR genes**

Four PPR genes (*CRR4*, *CRR21*, *CLB19*, *OTP82*) were selected for this study based on the number of editing site targets and their distribution in Brassicaceae species (Additional file 1). *CRR4* and *CRR21* are each required for editing a single site in *A. thaliana* chloroplasts. *CRR4* is required for editing *ndhD* C2 which is present in all species of Brassicaceae that were examined, and this condition is referred to as “homogenous” (Additional file 1). In contrast, the *CRR21* editing site target has been replaced by a genomic T and is absent in some crucifer species, and the editing site is “heterogeneous” in these taxa (Additional file 1). *CLB19* and *OTP82* each have two editing site targets in *A. thaliana* (Additional file 1). For the *CLB19* editing site targets, both editing sites are present in all species examined, while each of the *OTP82* sites is heterogeneous within the surveyed taxa (Additional file 1).

The extent of editing site conversion of the six editing sites associated with *CRR4*, *CRR21*, *CLB19*, and *OTP82* was determined in 21 species using bulk sequencing of cDNA (Table 1). The organisms included the 13 crucifer species with known plastid genomes plus species of agronomic importance (*Brassica oleracea, Lepidium sativum, Matthiola incana*, and *Raphanus sativus*) and representatives from diverse branches of the family ( *Thlaspi arvense*, *Iberis amara*, *Isatis tinctoria*, and *Hesperis matronalis*). Targets of CRR4 ( *ndhD* C2) and CLB19 ( *clpP* C559, *rpoA* C200) were determined to be edited in all species examined (Table 1). The percentage of edited transcripts for the CRR4 target, *ndhD* C2, varied dramatically from 10-80% (Table 1). The CLB19 target, *clpP* C559, was nearly fully edited in all species; however, the conversion of the second editing site target, *rpoA* C200, varied from 40-90% edited transcripts (Table 1). Thus, several species tolerate reduced editing of *rpoA* C200 in contrast to *clpP* C559.

**Table 1:** **Editing site conversion for*CRR4*,*CRR21*,*OTP82*and*CLB19*editing site targets**

	***CRR4***	***CRR21***	***OTP82***		***CLB19***	
	***ndhD***	***ndhD***	***ndhB***	***ndhG***	***clpP***	***rpoA***
*A. cordifolium*	50	100	100	90	90	80
*A. grandiflorum*	50	100	100	60	100	90
*A. hirsuta*	40	100	T	<10	100	60
*A. thaliana*	30	100	100	90	100	50
*B. oleracea*	60	100	T	90	100	80
*B. rapa*	30	100	T	90	100	90
*B. verna*	40	T	100	90	100	80
*C. bursa-pastoris*	80	100	100	80	100	80
*C. wallichii*	60	100	100	90	100	80
*D. nemorosa*	40	100	T	T	100	40
*L. maritima*	50	T	T	<10	100	50
*L. sativum*	50	100	100	90	100	90
*L. virginicum*	50	90	100	90	100	90
*M. incana*	70	T	100	70	90	90
*N. officinale*	10	100	90	90	100	70
*O. pumila*	70	100	100	80	100	90
*R. sativus*	50	100	T	90	100	80
*T. arvense*	20	T	T	90	100	90
*I. tinctora*	30	100	T	90	100	50
*H. matronalis*	60	100	T	90	100	60
*I. amara*	70	90	T	20	100	80

Data are expressed as percent of Cs converted to U. A “T” indicates that the plastid genome encodes a T at this position.

**Figure 1:**
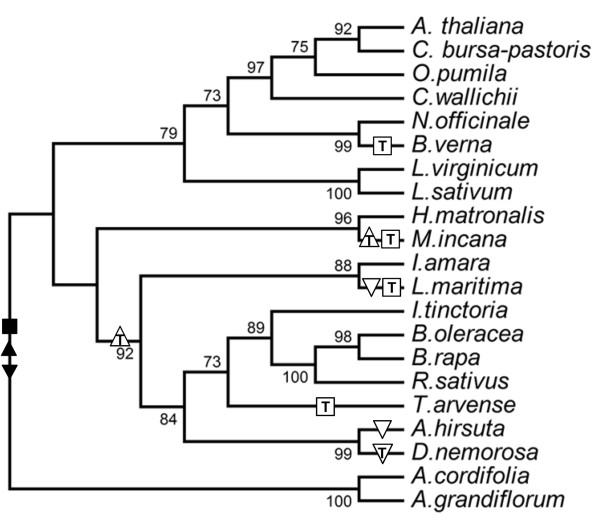
**Multiple independent losses of editing sites in Brassicaceae species.** The cladogram represents a ML phylogenetic tree built from *ndhB*, *ndhD*, *ndhG*, *rpoA*, and *clpP* sequences. aLRT branch values greater than 50 are indicated at each node. Editing activity for an individual editing site is indicated by the filled shapes: ■ for *ndhD* C383;▲ for *ndhB* C836; and ▼ for *ndhG* C50. Empty shapes indicate a lineage where loss of editing activity of a C is the most parsimonious explanation for lack of editing in extant species. Empty shapes with an inset T signal a loss of editing activity due to C → T genomic mutation.

Several Brassicaceae species lack editing sites targeted by CRR21 (*ndhD* C383) and OTP82 ( *ndhB* C836, *ndhG* C50) (Table 1). The CRR21 editing site target (*ndhD* C383) was either fully edited or the editing site was lost through substitution with a genomic T. This result is consistent with strong selection for complete RNA editing driven by a requirement for efficient chlororespiration [19]. A cladogram was constructed from *ndhBndhDndhG*, and *clpP* sequences to illustrate the evolutionary relationships of the 21 species (Figure 1). The tree topology is consistent with a larger cladogram generated using ITS sequences for a larger pool of Brassicaceae sequences [28]. The four losses of *ndhD* C383 are observed at different leaves on a cladogram (Figure 1), demonstrating that multiple, independent losses of editing sites occurred during the evolution of the Brassicaceae. These results expand on an earlier report of multiple independent losses of *matK* editing sites during plant evolution [29].

The evolution of OTP82 editing site targets was the most complex of the four examples. Two independent losses of the *ndhB* C836 editing site were observed (Figure 1). In one example, the *ndhB* C836 editing site was lost through a C to T genomic mutation in a node impacting many Brassicaceae species (Figure 1), while the second example occurred in the final branch leading to *M. incana*. For the *ndhG* C50 editing site, three species have either a genomic T that eliminates an editing site or retention of a C and loss of RNA editing (Figure 1). *L. maritima* retains the C at *ndhG* C50, but the transcripts exhibit less than 10% conversion to a T in the green leaf tissue examined (Table 1). In the branch leading to *A. hirsuta* and *D. nemorosa*, *ndhG* C50 was replaced by a genomic T in *D. nemorosa*, but remains as an unedited C in *A. hirsuta* (Table 1). RNA editing of *ndhG* C50 creates an F or L codon from an S codon in Brassicaceae; therefore, loss of editing would lead to radical amino acid substitutions in *A. hirsuta* and *L maritima*.

*Cis*-elements for chloroplast editing sites are generally considered to be within nucleotides −20 to +5 relative to the edited C [30-32]. The region comprising the *cis*-elements for the six editing sites examine in this study are nearly identical in the species examined (Additional file 2). In the lineages where an editing site is absent for CRR21 (*B. verna, M. incana, T. arvense, L. maritima*), the *cis*-elements for *ndhD* C383 show no unique substitutions compared with species that edit this site (Additional file 2). In the case of OTP82, nucleotide differences are present in the *A. hirsuta cis*-element for *ndhG* C50 and might interfere with editing site recognition. However, the *cis*-elements are unchanged in *L. maritima* and in *I. amara*, yet the *ndhG* C50 editing site exhibits a highly reduced level of conversion in these species (Table 1, Additional file 2). Editing is not required at the other *OTP82* editing site ( *ndhB* C836) as a result of a genomic T in that position. Thus, the inability to edit *ndhB* C50 in these species is probably the result of a loss of editing activity rather than a change in the recognition of the *cis*-element.

### The evolution of PPR genes in plants lacking an editing target

**Figure 2:**
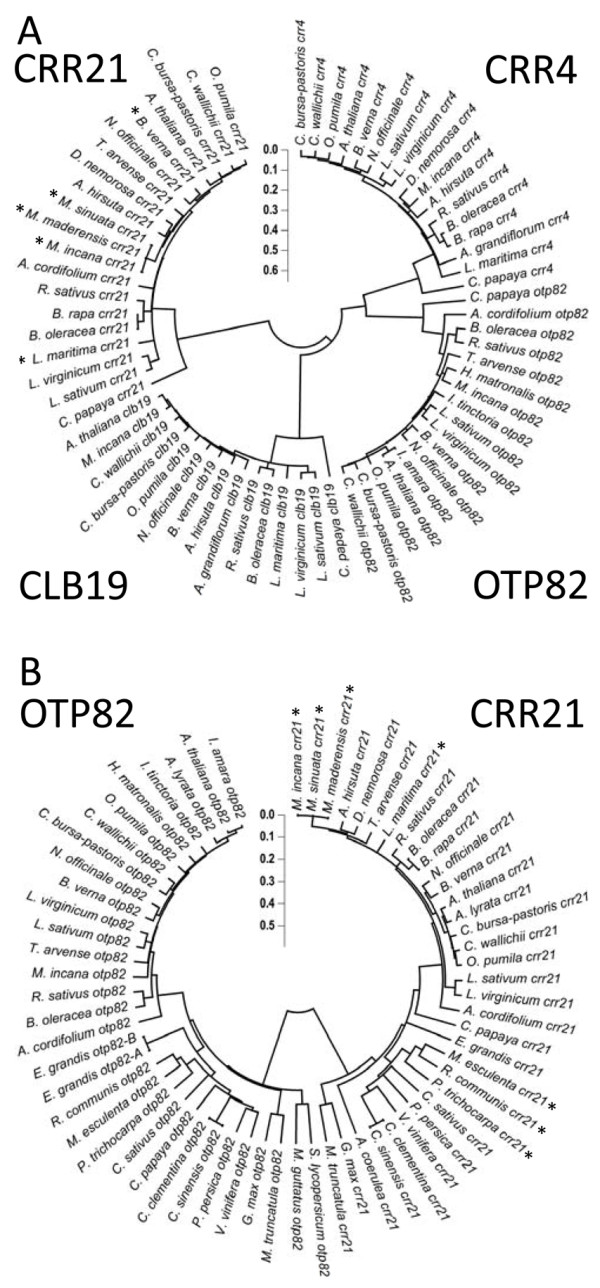
**Cladograms demonstrate orthology of PPR sequences.** ( **A**) A NJ tree built from translated *CRR4*, *CRR21*, *CLB19*, and *OTP82* genes sequenced from Brassicaceae species along with orthologs identified in a non-Brassicaceae member *Carica papaya* from Phytozome v7.0. ( **B**) A NJ tree built from *CRR21* and *OTP82* nucleotide sequences from dicots available in nucleotide databases and sequenced in this study. The NJ tree distance using the Maximum Composite Likelihood nucleotide substitution model is indicated by the scale. ( **A, B**) Accessions for species that lack the cognate editing site are indicated by an *. Amino acids used to construct trees align to the following positions of the current gene models in Arabidopsis: *CRR4* (33–446); *CRR21* (38–620); *CLB19* (67–467); and *OTP82* (72–525).

Sequences for putative orthologs to *CRR4*, *CRR21*, *CLB19*, and *OTP82* were obtained for representative Brassicaceae species through PCR and bulk sequencing (Additional file 3). Each putative PPR gene shares the same basic architecture in terms of number and order of PPR repeats (data not shown). An unrooted NJ phylogenetic tree built using the sequences indicates that each of the four PPR genes forms its own clade, and these genes are apparently orthologous (Figure 2).

**Figure 3:**
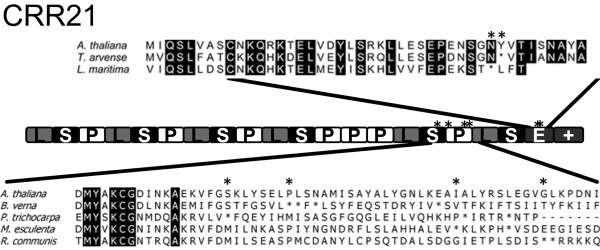
**Truncated putative orthologs of*CRR21 *in*B. verna*,*L. maritima*,*T. arvense*,*M. esculenta, R. communis,*and*P. trichocarpa*.** A cartoon highlights the relative position of truncations by * and the PPRs are represented by a series of boxes. The translated amino acid sequence is shown for two regions above and below the boxes representing the PPR genes. For the amino acid translation of the *CRR21* gene, in frame stop codons in the C-terminus and 3’UTR are represented with *. PLS designations are based on the PPR motifs previously described [1].

Several species in the Brassicaceae have lost editing targets for CRR21 or OTP82 (Figure 1). Putative orthologs for *CRR21* were detected in Brassicaceae species that lack the known target for this gene (Figure 3). The *A.* thaliana *CRR21* gene contains 19 PPR repeats and E and E + domains. Truncated *CRR21* genes were detected in three species: *B. verna* was truncated in PPR repeat 17; and both *L. maritim*a and *T. arvense* were truncated in the middle of the E domain (Figure 3). These truncated genes are known to be expressed as mRNAs since they were amplified as cDNAs by 3’-RACE. Since plants have frequently undergone genome or gene duplication, it is possible that additional full length copies of the *CRR21* gene might be present in these species. Based on PCR and 3’-RACE, the truncated forms of *CRR21* represent the only intact *CRR21* orthologs that could be detected in these species (see Additional file 4 for more information).

Since these taxa are separated on distinct branches (Figure 1), these results indicate that multiple independent truncation of the *CRR21* gene has occurred in species that have lost the editing site target. Remnants of the full E domain are easily detectable in *T. arvense*; however, the sequences derived from the E domain are not evident in 3’ UTR sequences from *B. verna* and *L. maritima* (Figure 3). Thus, the truncation in *T. arvense* is probably a more recent event. Recent loss of an editing target is also likely for three *Matthiola* species that have full length *CRR21* putative orthologs (Table 2). A fourth *Matthiola* species examined, *Matthiola longipetala*, is capable of editing *ndhD* C383, suggesting that the loss of editing target has occurred recently in the genus (data not shown).

**Table 2:** **Conservation of*CRR21*genes orphaned through loss of editing site target**

**Species**	***ndhD* 383**	***CRR21***	**dN**	**dS**	**dN/dS**	**dN/dS ≠ 1**
*Matthiola incana*	T	Full length	0.0206	0.169	0.122	p = 1.48e-16
*Matthiola sinuata*	T	Full length	0.0206	0.169	0.122	p = 1.48e-16
*Matthiola maderensis*	T	Full length	0.0206	0.169	0.122	p = 1.48e-16
*Thlaspi arvense*	T	Trunc. E	0.0145	0.183	0.079	p = 4.22e-20
*Lobularia maritima*	T	Trunc. E	0.0662	0.527	0.126	p = 9.47e-40
*Barbarea verna*	T	Trunc. R17	0.0079	0.055	0.246	p = 3.01e-06

The ratio of non-synonymous to synonymous substitutions, dN/dS, is a fundamental measure of the type and strength of selection that a protein coding gene has experienced [33]. Genes under strong negative selection exhibit small dN/dS values (<<1.0); this results from very few nucleotide substitutions that are expressed as amino acid substitutions. A dN/dS value of 1 indicates neutral selection, and dN/dS values greater than 1 indicate positive selection. The dN/dS values for the putative orthologs of *CRR21* in species that have lost editing targets are much less than 1, and are shown in Table 2. Orphaned *CRR21* genes, including both the truncated and the full length examples, are maintained under strong negative selection.

Sequences from a wider taxon sampling were examined to identify whether truncation of *CRR21* genes is a common feature or restricted to the Brassicaceae. Additional *CRR21* and *OTP82* genes were identified through Phytozome 7.0 in the sequenced genomes of *M. esculenta, R. communis, P. trichocarpa, M. truncatula, G. max, C. sativus, P. persica, A. lyrata, C. papaya, C. sinensis, C. clementine, E. grandis, V. vinifera, M. guttatus, and A. coerulea*. Genes were considered to be putative orthologs if they had greater than 50% sequence identity, were reciprocal best hits to Arabidopsis PPR proteins by TBLASTN, and formed a distinct clade on a NJ tree with the Brassicaceae members (Figure 2). Truncated forms of *CRR21* were detected in three species of the Malpighiales ( *M. esculenta*, *R. communis*, and *P. trichocarpa*), and no full length *CRR21* genes were found in any of these species (Figure 3). All three species have lost the *ndhG* C50 editing site by substitution with a genomic T (Figure 2), and loss of the editing site may have occurred in a common ancestor. *M. esculenta* and *R. communis* are both in the large Euphorbiaceae family, and the pairwise dN/dS value between *M. esculenta* and *R. communis CRR21* genes is 0.276, indicating strong negative selection of truncated genes. Thus, both truncation of CRR21 and loss of the *ndhD* C383 editing site target have occurred in additional examples in angiosperm evolution, and these truncated *CRR21* genes appear to be maintained under strong negative selection.

### Selective constraints for PPR domains varies based on differential selection in helix A and helix B motifs

**Figure 4:**
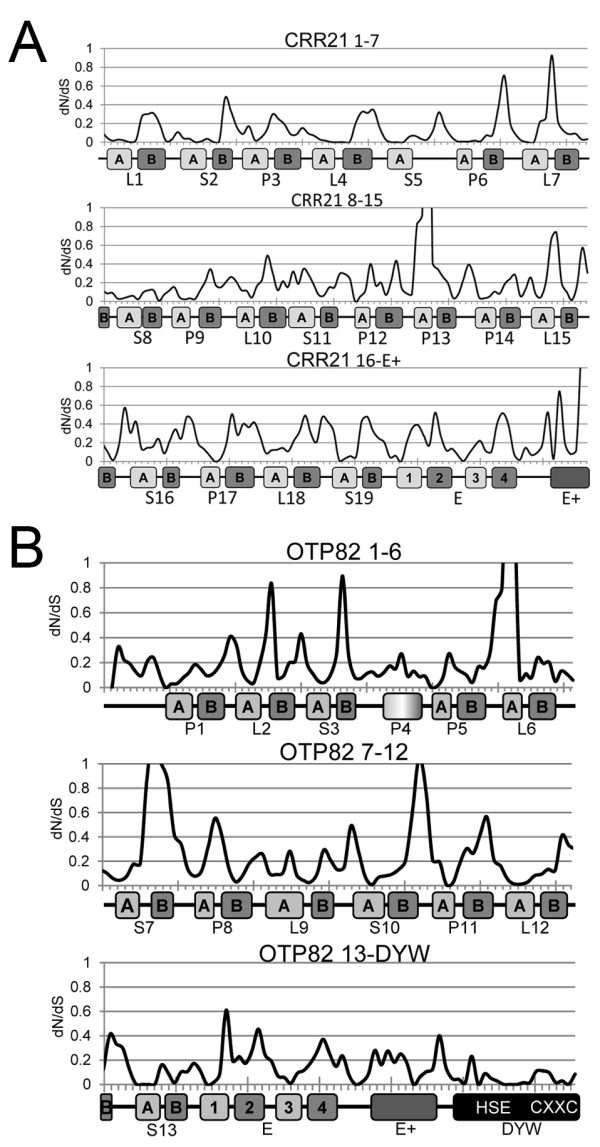
**Rate of evolution of the*CRR21*(A) and*OTP82*(B) genes in Brassicaceae species.** dN/dS for windows composed of 9 codons separated by a step of 3 codons were plotted versus the position of the midpoint nucleotide. Below the nucleotide positions the respective positions of predicted helices are indicated by boxes. Each PPR is labeled by the P, L, or S type PPR and the number of the repeat from the N-terminus. Boxes also indicate the E + and DYW domains.

Individual PPR domains are proposed to fold into two antiparallel helices (helix A and helix B) connected by a coil to create a structure with two faces [34-37]. In order to determine variation of selective pressure across the PPR genes, dN/dS was scanned across the gene sequences with a sliding window analysis of 9 codons with 3 codon increments for *CRR21* and *OTP82* (Figure 4) as well as *CRR4* and the mammalian PPR gene *PTCD3* (Additional file 5). These analyses show that very slowly evolving regions are most frequently located in specific PPR repeats, in the C-terminal region of the E domain, and in most of the DYW domain (Figure 4).

**Figure 5:**
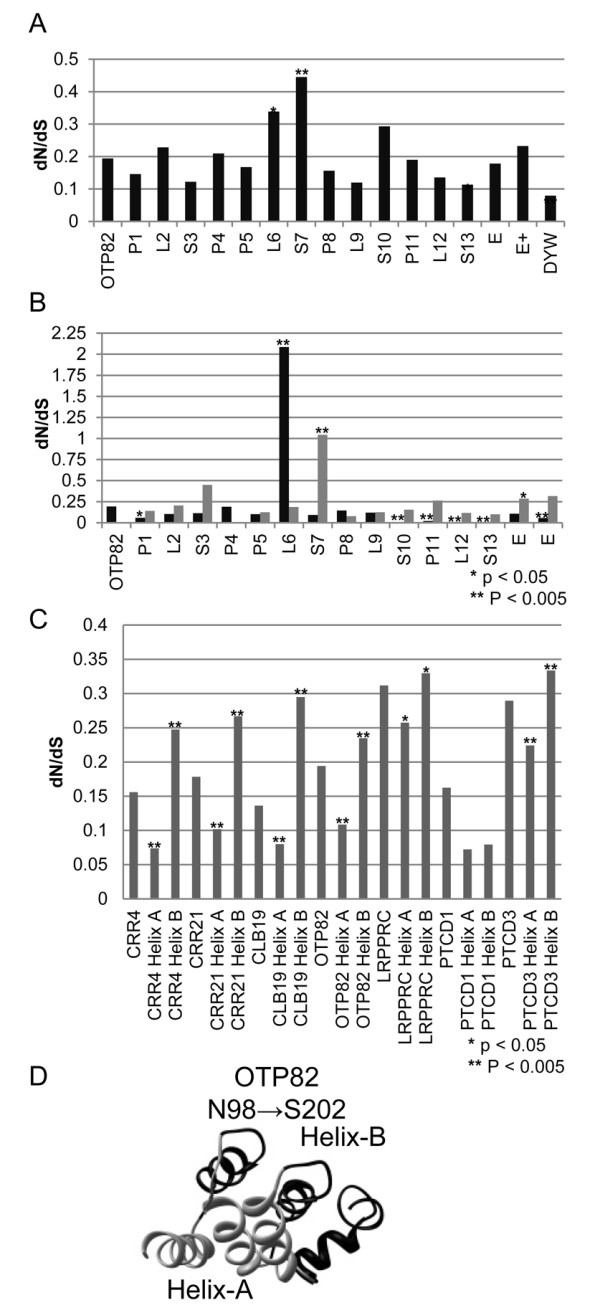
**Rate of evolution for individual PPR domains illustrated with*OTP82*.** Comparable data for *CRR4* and *CRR21* are shown in Additional file 4. ( **A**) dN/dS calculated for *OTP82* for each PPR domain, E, E+, and DYW domains. ( **A, B**) Each repeat is labeled 1 through 13 from the N terminus and P, L, and S domains are indicated [1]. (**B**) dN/dS versus the aligned PPR and each predicted helix. Black bars indicate values calculated from sequences encoding the entire gene, A helices, a single predicted helix for a PPR, and predicted helices 1 and 3 of the E domain. Grey bars indicate dN/dS values of predicted B-helices and helix 2 and 4 of the E domain. ( **A** and **B**) P-values were calculated to determine if computed dN/dS values are significantly different from the mean value for the entire aligned gene. The symbols * and ** indicate data points with p-values <0.05 and <0.005 respectively. **C**) Values for dN/dS are plotted calculated from 7 PPR genes. For each gene estimated dN/dS is shown for the entire aligned gene, concatenated A helices, and concatenated B-helices. P-values indicate the likelihood that predicted dN/dS values for one concatenated helix differs from the other. Symbols * and ** mark dN/dS estimates that have associated p-values <0.05 and <0.005 respectively. **D**) A structural prediction for a segment of the OTP82 protein. The image illustrates the inner and outer facing surfaces of the superhelical structure that are predicted to be created by the antiparallel helices of three PPR repeats. The region imaged corresponds to 3 repeats between residues N98 to S202 of OTP82.

The rates of evolution of each repeat and the C-terminal domains were determined for *OTP82* (Figure 5A). Repeats R6, R7, and R13 were found to have dN/dS values that differed significantly from the entire gene (Figure 5A). The DYW domain had the lowest dN/dS value of all domains examined with a value of 0.08 compared with the gene average of 0.19 (Figure 5A). All PPRs had dN/dS values less than 1 and are under strong negative selection (Figure 5A).

Since PPR proteins are thought to be composed of pairs of antiparallel helices [1,34-37], dN/dS values were determined for the A helices and B helices of the individual repeats for *OTP82* (Figure 5B). The A helices generally exhibit significantly smaller dN/dS values than the gene as a whole, and this is especially notable in helix A of PPR repeats P1, S10, P11, L12, and S13 (Figure 5B). The third helix of the E domain also had a significantly lower dN/dS value than the mean for the gene (Figure 5B). Helices that have significantly higher dN/dS values than the entire gene include helix A of L6 as well as helix B of S7 and helix 2 of the E domain (Figure 5B).

In order to determine if the difference in selective pressure on helix A and helix B is a general feature of PPR genes, the evolution of A and B helices was compared in the four Brassicaceae PPR genes (*CRR4*, *CRR21*, *CLB19*, and *OTP82*) and three well studied mammalian PPR genes ( *LRPPRC, PTCD1, and PTCD3*). For each PPR gene, sequences encoding helix A were selected and concatenated into a single sequence alignment file containing only helix A sequences. Helix B sequences from the second helix of each PPR repeat were treated similarly. The dN/dS ratio was determined for helix A and helix B sequences for each of the genes surveyed (Figure 5C). For six of seven PPR genes examined, helix A is under greater negative selective pressure than helix B (Figure 5C).

## Discussion

### PPR gene and target co-evolution

Our analyses demonstrate that RNA editing constrains both the evolution of a PPR gene responsible for editing as well as the *cis*-element recognized by the PPR protein. Earlier work established that *cis-*elements in angiosperm chloroplasts recognized by PPR proteins are constrained, and that the loss of an editing requirement by substitution of an edited C with a genomic T relaxed the constraints on *cis*-element evolution [27,38,39]. The results of this study extend these observations through comparison of the evolutionary changes in PPR genes when editing sites are maintained or are lost in evolution. PPR genes such as *CRR4* and *CLB19* that have evolved in the context of a homogenous requirement for editing are maintained under strong negative selection and gene architecture is stable.

By contrast, PPR genes such as *CRR21* and *OTP82* have evolved in an environment where the requirement to edit has changed. Loss of an editing target has previously been shown to correlate with conversion of *CRR4* into a pseudogene in *Medicago truncatula*[27]. As a contrasting example, this study has shown that the *CRR21* gene has been maintained intact after a recent editing site loss, as observed in *Matthiola* species. Additional examples of editing site loss are observed for the *OTP82* gene, which is required for the conversion of two editing site targets. The *OTP82* gene is maintained under strong negative selection after loss of the *ndhB* C836 editing site by substitution with a genomic T in *T. arvenseI*. *tinctoriaR. sativusB. oleracea*, and *B. rapa* (Figure 1), presumably because *OTP82* is still required for a second editing activity at *ndhG* C50 in all of these species.

A third consequence of editing site loss is the observation of gene truncation with *CRR21*. The *ndhD* C383 editing site has been lost by substitution with a genomic T in *T. arvenseB. vernaL. maritima, P. trichocarpaR. communis*, and *M. esculenta*. Orthologs of *CRR21* are present in all six species, but have truncated C-terminal domains in or near repeat 17 or in the middle of the E domain (Figure 3). All known chloroplast PPR proteins that are required for editing site conversion have an intact E domain, and the C-terminal domains of CRR4 have been shown to be critical for editing functions in *A. thaliana*[19]; thus, there is strong evidence that the E domain is required for a functional editing PPR protein.

We observed at least three independent C-terminal truncations of *CRR21* genes in the Brassicaceae, yet these orphaned *CRR21* genes are maintained under strong negative selection. Several observations suggest that these truncated *CRR21* genes are not a first step in pseudogene formation. First, the dN/dS values are very small within the coding sequences of the truncated PPR genes, and range from 0.079 to 0.246 (Table 2). Second, examination of the C-terminal regions beyond the truncation site indicates that these sequences that now comprise the 3’UTR have experienced very high levels indels and nucleotide substitutions (Figure 3). This is consistent with a loss of selection in the 3’ UTR that was previously maintained as coding sequence. Only the 3’UTR of the truncated *CRR21* in *T. arvense* bears recognizable similarity to the C-terminal coding sequences of *A. thaliana*, and this may reflect a recent truncation event. Finally, analysis of *CRR21* genes in sequenced genomes that have a genomic T at *ndhD* C383 demonstrates that *CRR21* truncation is observed in three additional species of the Malpighiales, and that these genes are maintained under strong negative selection for what appears to be an extended period of evolution.

The maintenance of PPR genes responsible for editing and the *cis*-elements of an editing site appear to involve mutual selective pressure on both components. Loss of an editing site by C-to-T mutation frequently correlates with pseudogene formation or structural changes in the PPR gene. Loss of PPR gene function may result in an unedited C at an editing site.

### Molecular evolution of PPR motifs reveals differential selective pressure on helix A and helix B motifs

PPR proteins bind RNA in a sequence specific manner, and recent molecular phylogenetic and modeling analyses have proposed that residues in and near the A helices may be directly involved in RNA binding [35]. Our analyses demonstrate that selective pressure varies across the gene sequences in four plant PPR genes and two mammalian PPR genes, such that helix A sequences are highly restricted in evolution and that helix B sequences are less constrained. Since both the PPR genes and the corresponding *cis*-elements are under strong negative selection, these results suggest that amino acid residues in the A helices are critical for PPR function. PPR proteins have sequence and predicted secondary structural similarities to the tetratricopeptide repeat (TPR) proteins, and individual PPR domains are proposed to fold into a pair of antiparallel helices (helix A and helix B) connected by a coil to create a structure with two faces (Figure 5D) [34,36]. Thus, helix A amino acid residues may be involved in RNA binding or substrate recognition.

Recent molecular phylogenetic analyses and protein modeling of PPR genes in a subclass of genes known as Restorer of fertility genes ( *Rf* type PPR) provide an interesting perspective and contrast to these results [35]. *Rf* type PPR genes restore fertility to plants that would otherwise exhibit cytoplasmic male sterility ( *cms*) [6,40,41]. The *Rf* type PPR genes function by recognizing novel mitochondrial transcripts that interfere with pollen formation, and the *Rf* genes and mitochondrial transcripts co-evolve in a nuclear-organellar arms race of diversifying selection [35]. Some amino acid residues in or near helix A sequences in these *Rf* genes are under diversifying (positive) selection, apparently in response to changes in the target RNA sequences that may be driven by mutation and recombination. Thus, these residues may be directly involved in RNA binding.

By contrast, the editing-related PPR genes examined in this study have largely evolved in the presence of stable *cis*-elements and the corresponding PPR genes are under purifying (negative) selection. In this work, helix A sequences have been shown to be highly constrained, and these results taken together with the molecular phylogenetic analysis of the Rf type PPR genes strongly suggest that helix A residues may be involved in RNA binding and substrate recognition.

## Conclusion

Our results demonstrate that loss of chloroplast editing sites has several potential outcomes for PPR genes. First, the cognate PPR gene can become a pseudogene. Second, no change in the PPR gene may be observed and the PPR gene architecture is under negative selection for an alternate function. Third, truncations that remove C-terminal domains occurred independently and in similar regions of the *CRR21* gene in different lineages. The truncated *CRR21* genes are maintained under strong negative selection, and this suggests that they may be maintained for an unrecognized function that is potentially distinct from RNA editing.

Selective pressure differs between individual PPR repeats and within helix A and helix B regions of the PPR repeats. The A helices are under exceptionally strong negative selection compared to B helices. The increased selection strongly suggests that there is a critical role for residues that comprise the helix A, possibly in binding RNA.

## Methods

### Seeds for plant materials

*Arabidopsis thaliana* col-0 seeds were obtained from Lehle Seeds (Round Rock, TX). Seeds of strains with sequenced chloroplast genomes were obtained from the RIKEN BRC Experimental Plant Division SENDAI Arabidopsis Seed Stock Center (SASSC) for *Arabis hirsuta* (SJO02300), *Crucihimalaya wallichii* (SJS00500), *Draba nemorosa* (SJO02100), *Lepidium virginicum* (SJO02600), and *Olimarabidopsis pumila* (SJS00200). The following seeds were obtained from the KEW Gardens Millennium Seed Bank Project: *Capsella bursa-pastoris* (Serial # 0051714); *Nasturtium officinale* (Serial #0200327); *Matthiola sinuata* (Serial #0070591). Seeds for *Matthiola longipetala* (PI 633271), *Matthiola maderensis* (PI 650275), *Iberis amara* (PI 633243), *Thlaspi arvense* (Ames 29531), *Hesperis matronalis* (PI 586611), and *Istatis tinctoria* (Ames 21376) were obtained from the United States Department of Agriculture ARS-GRIN. *Raphanus sativum* (winter radish) seed came from Samen-Kӧller, Sudtirolerplatz 1, Graz, Austria. *Brassica oleracea* var. viridis (collards) seeds were purchased from the Ferry-Morse Seed. Co. (Fulton, KY). Seeds were purchased from Johnny’s Selected Seeds (Winslow, ME) for *Matthiola incana* (Stocks Giant Excelsior Mix), *Brassica rapa* (Hybrid Chinese cabbage, Napa), *Nasturtium officinale* (watercress), *Barbarea verna* (upland cress), and *Lepidium sativum* (curly cress). *Aethionema grandiflorum* seed was purchased from Hazzard’s Wholesale Seeds (Deford, Michigan). Seeds for *Lobularia maritima* ‘New Carpet of Snow’ and *Aethionema cordifolium* were acquired from Hardyplants.com (Apple Valley, MN).

### PCR amplification and bulk sequencing

Genomic DNA was isolated from green, true leaves using a CTAB method [42]. We used Fermentas DreamTaq Green DNA Polymerase from ThermoFisher (Waltham, MA), 2 ng gDNA, and PCR primers listed in Additional file 6 for PCR amplifications. Ambion Tri-Reagent from Life Technologies (Carlsbad, California) was used to isolate RNA from green, true leaves. We used Fermentas Maxima Reverse Transcriptase from ThermoFisher (Waltham, MA), Random Primers from Promega (Madison, WI), and 1 μg of total RNA to create cDNAs. RT-PCR products were then generated using 2μL of cDNA per 50 μL reaction and appropriate primers listed in Additional file 6**.** Bulk sequencing was performed at the University of California, Berkeley DNA Sequencing Facility using primers listed in Additional file 6. Quantification of editing was performed using the raw trace file. The Peak height for C and T at the editing site position was measured and percent editing (%T) calculated. Percent editing was rounded to the nearest 10%.

### 3’-race protocol

We used Fermentas Maxima Reverse Transcriptase from ThermoFisher (Waltham, MA) to create cDNAs using 1 μg of total RNA and primer polyT_KS (Additional file 6). We then used the generated cDNA as template in a PCR reaction using a site specific primer for the PPR and the KS primer from Additional file 6. PCR product from the previous amplification was diluted 1:200 and 1 μL used as template in a second PCR using a nested primer. Products were the sequenced using appropriate nested primers from Additional file 5.

### Chromosome walking for 5’ ends

In order to get 5’ flanking sequences for several PPR genes we used a slightly modified the SiteFinding-PCR protocol for chromosome walking [43]. Modifications included using modified primers Sitefinder1, SFP1 and SFP2 for respective random priming, PCR round #1, and PCR round #2. For PCR rounds #1 and #2 nested primers were used listed in Additional file 6. For round #2 two different nested primers were used to yield slightly different size PCR products so bands resulting from specific priming could be easily distinguished

### Determination of putative orthologs for PPR genes in brassicaceae

Sequences obtained from PCR and bulk sequencing in Brassicaceae species were inspected for large intact reading frames. Translated ORFs were aligned with Arabidopsis PPR gene sequences using ClustalW. Nucleotide sequences typically shared 70-90% identity with Arabidopsis gene sequences and greater amino acid identity. A neighbor-joining (NJ) phylogenetic tree was constructed using Mega5 with the pairwise deletion parameter selected for gaps/missing data as well as the nucleotide substitution model parameter selected for the Maximum Composite Likelihood Model. Phylogenetic gene trees had similar topology as species trees constructed by [28] and sequences from each PPR formed four distinct clades.

### Identification of putative orthologous sequences in sequenced genomes

*A. thaliana* amino acid sequences from the CRR21 protein model AT5G55740.1 and the OTP82 protein model AT1G08070.1 were queried using TBLATN to all 15 dicot genomes excluding *A. thaliana* ( *Manihot esculenta, Ricinus communis, Populus trichocarpa, Medicago truncatula, Glycine max, Cucumis sativus, Prunus persica, Arabidopsis lyrata, Carica papaya, Citrus sinensis, Citrus clementine, Eucalyptus grandis, Vitis vinifera, Mimulus guttatus, and Aquilegia coerulea*) deposited in Phytozome v7.0 at http://www.phytozome.net/. All queries resulted in a single hit with E values of 0 except for in *E. grandis* for OTP82 where there were two hits to different parts of the genome. A putative ortholog for OTP82 was also found in *Solanum lycopersicum* sequences available in the NCBI databases using the same methodology. Hits had amino acid identity greater than 50% and were queried using TBLASTN back to *A. thaliana*. If the reciprocal best hit was to the initial queried gene sequence the gene was considered to be putatively orthologous. The putative orthologs for *CRR21* and *OTP82* were aligned along with Brassicaceae sequences generated in this survey using ClustalW. A neighbor-joining (NJ) phylogenetic tree was constructed using Mega5 which separated *CRR21* and *OTP82* sequences into distinct clades. Mammalian putative orthologs to human *LRPPRC*, *PTCD1*, and *PTCD3* were identified using TBLASTN and reciprocal best hit using the nucleotide sequence collection in the NCBI databases http://www.ncbi.nlm.nih.gov/. Like the plant sequences putative orthologs had E-values of 0 using TBLASTN and only single hits were found. *LRPPRC* sequences analyzed were from *Ailuropoda melanoleuca*, *Bos taurus*, *Callithrix jacchus*, *Equus caballus*, *Homo sapiens*, *Macaca mulatta*, *Mus musculus*, *Oryctolagus cuniculus*, and *Pan troglodytes*. *Ptcda1* putative orthologs were analyzed from sequences from *Monodelphis domestica*, *A. melanoleuca*, *B. taurus*, *C. jacchus*, *E. caballus*, *M. mulatta*, *O. cuniculus*, *P. troglodytes*, *H. sapiens*, *Pongo abelii*, *Rattus norvegicus*, and *Sus scrofa*. *Ptcda3* sequences were from *M. domestica*, *A. melanoleuca*, *B. taurus*, *C. jacchus*, *E. caballus*, *H. sapiens*, *M. mulatta*, *M. musculus*, *O. cuniculus*, *P. troglodytes*, *P. abelii*, *R. norvegicus*, and *S. scrofa*.

### Calculation of dN/dS

Sequences generated in this study and those available in the GenBank databases were used to calculate dN/dS. GenBank accession numbers were assigned to our PPR sequences are listed in Additional file 3. Sequences were aligned using the Clustal W aligner using the default settings within the program BioEdit 7.0.9.0 [44] from http://www.mbio.ncsu.edu/bioedit/bioedit.html. A maximum likelihood (ML) phylogenetic gene tree was generated using sequences from each aligned PPR with PHYML using the GTR parameter for the nucleotide substitution model [45]. dN/dS values were calculated from an alignment and phylogentic tree using the program codeml of the PAML suite of programs [46]. For scanning window analysis, a program took a sequence alignment of an entire gene and parsed it into windows of 27 nucleotides with 9 nucleotide increments. Each window of aligned sequences was input into codeml with the phylogenetic tree for the entire gene. For dN/dS analysis of individual PPR domains, repeats were defined using the Uniprot model for each gene http://www.uniprot.org/uniprot. For calculations of dN/dS from individual helix A or helix B, secondary structure predictions were made using HHpred and sequences trimmed appropriately [47]. Model 0 of codeml was used to estimate mean dN/dS given the phylogeny. To test if PPRs were significantly different than the average dN/dS for the gene, the parameter ω was set to the average dN/dS calculated for the entire gene and log likelihood was generated for the model. A mean likelihood test was performed to generate p values. To test if dN/dS for helix A is significantly different than helix B, mean dN/dS for the tree was estimated for concatenated helix A and helix B encoding sequences. Then ω was set as the generated value for the opposite helix to get likelihoods for those models. Finally, to test purifying selection for an individual branch for *CRR21*, we used two models; model 2a which sets the model parameter to 2 and fix_omega to 0; and model 2b which sets the model parameter to 2, fix_omega to 1 and ω = 1.

### Protein modeling

We developed structural models for CRR4, CRR21, CLB19, and OTP82 to examine conserved structural features of these proteins. Secondary structural predictions were initially made using HHpred program and generally predicted two anti-parallel helices per PPR [47]. The prediction is in line with consensus model for PPR secondary structure [1,35,48]. Secondary and tertiary structural predictions were performed with I-Tasser software that identifies template proteins with similar folds from the PDB library, and predicts a model through a series of protein-modeling algorithms [49]. The E domain was predicted to fold into two sets of antiparallel helices (data not shown), similar to two additional PPRs. Structures were visualized using PyMOL and secondary structure features highlighted.

### Species tree

A Brassicaceae species tree was built using chloroplast cDNA sequences obtained for *ndhBndhDndhGrpoA*, and *clpP*. Sequences were aligned using ClustalW. A ML tree was constructed using PHYML and the GTR nucleotide substitution model. The tree was visualized using MEGA5 [50] and aLRT branch-support numbers [51] are displayed on each branch.

## Authors' contributions

MLH and RMM designed the study and drafted the manuscript. MLH obtained plant materials and developed Methods used in this survey. MLH and KG performed the laboratory work. All authors contributed to the writing of the paper and approve the final version.

## Supplementary Material

Additional file 1 ** Distribution of putative editing sites in selected Brassicaceae linages.** A table depicts the nucleotide that aligns with an editing site in *A. thaliana* derived from chloroplast sequences for Brassicaceae species. Click here for file

Additional file 2 ** Alignment of*cis*-elements for CRR4, CRR21, CLB19, and OTP82.** A figure illustrates nucleotide sequences around editing sites targeted by four PPR proteins. Each sequence represents 20 nucleotides upstream and 5 nucleotides downstream of the editing site. The editing sites are indicated capitalized characters. Nucleotides that are 100% conserved are blocked in black. Click here for file

Additional file 3 ** Genebank accession numbers of analyzed sequences.** Genebank accession numbers for sequences of PPR genes determined by this work are provided in a single table. Click here for file

Additional file 4 ** Examination of species with truncated*CRR21*genes for potential duplicate copies of*CRR21.*** Click here for file

Additional file 5 ** Rate of evolution of the*CRR4 * genes in Brassicaceae and *PTCD3 *in mammals.** The dN/dS values for a 27nt window are plotted versus the midpoint position of each window for *CRR4 * (at top) and *PTCD3 * (at bottom). Below the nucleotide positions the respective positions of predicted helices are indicated by labeled boxes. Click here for file

Additional file 6 Primers used in this survey. Click here for file

## References

[B1] Lurin C Andrés C Aubourg S Bellaoui M Bitton F Bruyère C Caboche M Debast C Gualberto J Hoffmann B Genome-wide analysis of Arabidopsis pentatricopeptide repeat proteins reveals their essential role in organelle biogenesis Plant Cell 2004 16 8 2089 2103 10.1105/tpc.104.022236 15269332 PMC519200

[B2] Knoop V Rüdinger M DYW-type PPR proteins in a heterolobosean protist: plant RNA editing factors involved in an ancient horizontal gene transfer? FEBS Lett 2010 584 20 4287 4291 10.1016/j.febslet.2010.09.041 20888816

[B3] Iyer LM Zhang DP Rogozin IB Aravind L Evolution of the deaminase fold and multiple origins of eukaryotic editing and mutagenic nucleic acid deaminases from bacterial toxin systems Nucleic Acids Res 2011 39 22 9473 9497 10.1093/nar/gkr691 21890906 PMC3239186

[B4] Rüdinger M Polsakiewicz M Knoop V Organellar RNA editing and plant-specific extensions of pentatricopeptide repeat proteins in Jungermanniid but not in Marchantiid liverworts Mol Biol Evol 2008 25 7 1405 1414 10.1093/molbev/msn084 18400790

[B5] Salone V Rüdinger M Polsakiewicz M Hoffmann B Groth-Malonek M Szurek B Small I Knoop V Lurin C A hypothesis on the identification of the editing enzyme in plant organelles FEBS Lett 2007 581 22 4132 4138 10.1016/j.febslet.2007.07.075 17707818

[B6] Bentolila S Alfonso AA Hanson MR A pentatricopeptide repeat-containing gene restores fertility to cytoplasmic male-sterile plants Proc Natl Acad Sci U S A 2002 99 16 10887 10892 10.1073/pnas.102301599 12136123 PMC125068

[B7] Fisk DG Walker MB Barkan A Molecular cloning of the maize gene CRP1 reveals similarity between regulators of mitochondrial and chloroplast gene expression Embo J 1999 18 9 2621 2630 10.1093/emboj/18.9.2621 10228173 PMC1171341

[B8] Kotera E Tasaka M Shikanai T A pentatricopeptide repeat protein is essential for RNA editing in chloroplasts Nature 2005 433 7023 326 330 10.1038/nature03229 15662426

[B9] Mancebo R Zhou XL Shillinglaw W Henzel W MacDonald PM BSF binds specifically to the bicoid mRNA 3 ' untranslated region and contributes to stabilization of bicoid mRNA Mol Cell Biol 2001 21 10 3462 3471 10.1128/MCB.21.10.3462-3471.2001 11313472 PMC100268

[B10] Manthey GM Mcewen JE The Product of the Nuclear Gene PET309 Is Required for Translation of Mature Messenger-Rna and Stability or Production of Intron-Containing Rnas Derived from the Mitochondrial COX1 Locus of Saccharomyces-Cerevisiae Embo J 1995 14 16 4031 4043 7664742 10.1002/j.1460-2075.1995.tb00074.x PMC394481

[B11] Xu F Morin C Mitchell G Ackerley C Robinson BH The role of the LRPPRC (leucine-rich pentatricopeptide repeat cassette) gene in cytochrome oxidase assembly: mutation causes lowered levels of COX (cytochrome c oxidase) I and COX III mRNA Biochem J 2004 382 Pt 1 331 336 15139850 10.1042/BJ20040469 PMC1133946

[B12] Covello PS Gray MW RNA editing in plant mitochondria Nature 1989 341 6243 662 666 10.1038/341662a0 2552326

[B13] Maier RM Neckermann K Hoch B Akhmedov NB Kossel H Identification of Editing Positions in the Ndhb Transcript from Maize Chloroplasts Reveals Sequence Similarities between Editing Sites of Chloroplasts and Plant-Mitochondria Nucleic Acids Res 1992 20 23 6189 6194 10.1093/nar/20.23.6189 1282235 PMC334503

[B14] Covello PS Gray MW On the evolution of RNA editing Trends Genet 1993 9 8 265 268 10.1016/0168-9525(93)90011-6 8379005

[B15] Cai W Ji D Peng L Guo J Ma J Zou M Lu C Zhang L LPA66 is required for editing psbF chloroplast transcripts in Arabidopsis Plant Physiol 2009 150 3 1260 1271 10.1104/pp.109.136812 19448041 PMC2705037

[B16] Chateigner-Boutin AL Ramos-Vega M Guevara-Garcia A Andrés C de la Luz Gutiérrez-Nava M Cantero A Delannoy E Jiménez LF Lurin C Small I CLB19, a pentatricopeptide repeat protein required for editing of rpoA and clpP chloroplast transcripts Plant J 2008 56 4 590 602 10.1111/j.1365-313X.2008.03634.x 18657233

[B17] Hammani K Okuda K Tanz SK Chateigner-Boutin AL Shikanai T Small I A study of new Arabidopsis chloroplast RNA editing mutants reveals general features of editing factors and their target sites Plant Cell 2009 21 11 3686 3699 10.1105/tpc.109.071472 19934379 PMC2798323

[B18] Okuda K Hammani K Tanz SK Peng LW Fukao Y Myouga F Motohashi R Shinozaki K Small I Shikanai T The pentatricopeptide repeat protein OTP82 is required for RNA editing of plastid ndhB and ndhG transcripts Plant J 2010 61 2 339 349 19845878 10.1111/j.1365-313X.2009.04059.x

[B19] Okuda K Myouga F Motohashi R Shinozaki K Shikanai T Conserved domain structure of pentatricopeptide repeat proteins involved in chloroplast RNA editing Proc Natl Acad Sci U S A 2007 104 19 8178 8183 10.1073/pnas.0700865104 17483454 PMC1876591

[B20] Robbins JC Heller WP Hanson MR A comparative genomics approach identifies a PPR-DYW protein that is essential for C-to-U editing of the Arabidopsis chloroplast accD transcript RNA 2009 15 6 1142 1153 10.1261/rna.1533909 19395655 PMC2685521

[B21] Zhou W Cheng Y Yap A Chateigner-Boutin AL Delannoy E Hammani K Small I Huang J The Arabidopsis gene YS1 encoding a DYW protein is required for editing of rpoB transcripts and the rapid development of chloroplasts during early growth Plant J 2008 58 1 82 96 19054358 10.1111/j.1365-313X.2008.03766.x

[B22] Okuda K Habata Y Kobayashi Y Shikanai T Amino acid sequence variations in Nicotiana CRR4 orthologs determine the species-specific efficiency of RNA editing in plastids Nucleic Acids Res 2008 36 19 6155 6164 10.1093/nar/gkn629 18824480 PMC2577327

[B23] Verbitskiy D van der Merwe JA Zehrmann A Härtel B Takenaka M The E-class PPR Protein MEF3 of Arabidopsis thaliana can Function in Mitochondrial RNA editing also with an Additional DYW Domain Plant Cell Physiol 2012 53 2 358 367 10.1093/pcp/pcr182 22186180

[B24] Verbitskiy D Zehrmann A van der Merwe JA Brennicke A Takenaka M The PPR protein encoded by the LOVASTATIN INSENSITIVE 1 gene is involved in RNA editing at three sites in mitochondria of Arabidopsis thaliana Plant J 2010 61 3 446 455 10.1111/j.1365-313X.2009.04076.x 19919573

[B25] Zehrmann A Verbitskiy D Härtel B Brennicke A Takenaka M PPR proteins network as site-specific RNA editing factors in plant organelles RNA Biol 2011 8 1 67 70 10.4161/rna.8.1.14298 21289490

[B26] Tillich M Poltnigg P Kushnir S Schmitz-Linneweber C Maintenance of plastid RNA editing activities independently of their target sites Embo Rep 2006 7 3 308 313 10.1038/sj.embor.7400619 16415790 PMC1456890

[B27] Hayes ML Mulligan RM Pentatricopeptide repeat proteins constrain genome evolution in chloroplasts Mol Biol Evol 2011 28 7 2029 2039 10.1093/molbev/msr023 21263042

[B28] Bailey CD Koch MA Mayer M Mummenhoff K O'Kane SL Warwick SI Windham MD Al-Shehbaz IA Toward a global phylogeny of the Brassicaceae Mol Biol Evol 2006 23 11 2142 2160 10.1093/molbev/msl087 16916944

[B29] Tillich M Le Sy V Schulerowitz K von Haeseler A Maier UG Schmitz-Linneweber C Loss of matK RNA editing in seed plant chloroplasts Bmc Evol Biol 2009 9 201 10.1186/1471-2148-9-201 19678945 PMC2744683

[B30] Chaudhuri S Maliga P Sequences directing C to U editing of the plastid psbL mRNA are located within a 22 nucleotide segment spanning the editing site Embo J 1996 15 21 5958 5964 8918473 PMC452380

[B31] Hayes ML Hanson MR Identification of a sequence motif critical for editing of a tobacco chloroplast transcript RNA 2007 13 2 281 288 17158709 10.1261/rna.295607 PMC1781371

[B32] Miyamoto T Obokata J Sugiura M Recognition of RNA editing sites is directed by unique proteins in chloroplasts: biochemical identification of cis-acting elements and trans-acting factors involved in RNA editing in tobacco and pea chloroplasts Mol Cell Biol 2002 22 19 6726 6734 10.1128/MCB.22.19.6726-6734.2002 12215530 PMC134032

[B33] Yang ZH Bielawski JP Statistical methods for detecting molecular adaptation Trends Ecol Evol 2000 15 12 496 503 10.1016/S0169-5347(00)01994-7 11114436 PMC7134603

[B34] Small ID Peeters N The PPR motif - a TPR-related motif prevalent in plant organellar proteins Trends Biochem Sci 2000 25 2 46 47 10664580 10.1016/s0968-0004(99)01520-0

[B35] Fujii S Bond CS Small ID Selection patterns on restorer-like genes reveal a conflict between nuclear and mitochondrial genomes throughout angiosperm evolution Proc Natl Acad Sci U S A 2011 108 4 1723 1728 10.1073/pnas.1007667108 21220331 PMC3029733

[B36] Delannoy E Stanley WA Bond CS Small ID Pentatricopeptide repeat (PPR) proteins as sequence-specificity factors in post-transcriptional processes in organelles Biochem Soc Trans 2007 35 1643 1647 10.1042/BST0351643 18031283

[B37] Ringel R Sologub M Morozov YI Litonin D Cramer P Temiakov D Structure of human mitochondrial RNA polymerase Nature 2011 478 7368 269 273 10.1038/nature10435 21947009

[B38] Hayes ML Hanson MR High conservation of a 5' element required for RNA editing of a C target in chloroplast psbE transcripts J Mol Evol 2008 67 3 233 245 10.1007/s00239-008-9101-9 18696032

[B39] Tillich M Funk HT Schmitz-Linneweber C Poltnigg P Sabater B Martin M Maier RM Editing of plastid RNA in Arabidopsis thaliana ecotypes Plant J 2005 43 5 708 715 10.1111/j.1365-313X.2005.02484.x 16115067

[B40] Kazama T Nakamura T Watanabe M Sugita M Toriyama K Suppression mechanism of mitochondrial ORF79 accumulation by Rf1 protein in BT-type cytoplasmic male sterile rice Plant Journal 2008 55 4 619 628 10.1111/j.1365-313X.2008.03529.x 18435825

[B41] Uyttewaal M Arnal N Quadrado M Martin-Canadell A Vrielynck N Hiard S Gherbi H Bendahmane A Budar F Mireau H Characterization of Raphanus sativus Pentatricopeptide Repeat Proteins Encoded by the Fertility Restorer Locus for Ogura Cytoplasmic Male Sterility Plant Cell 2008 20 12 3331 3345 10.1105/tpc.107.057208 19098270 PMC2630448

[B42] Rogers SO Bendich AJ Extraction of DNA from Milligram Amounts of Fresh, Herbarium and Mummified Plant-Tissues Plant Mol Biol 1985 5 2 69 76 10.1007/BF00020088 24306565

[B43] Tan GH Gao Y Shi M Zhang XY He SP Cheng ZL An CC SiteFinding-PCR: a simple and efficient PCR method for chromosome walking Nucleic Acids Res 2005 33 13 e122 10.1093/nar/gni124 16077029 PMC1182332

[B44] Hall TA BioEdit: a user-friendly biological sequence alignment editor and analysis program for Windows 95/98/NT Nucleic Acids Symposium Series (1999) 1999 41 41 95 98

[B45] Guindon S Gascuel O A simple, fast, and accurate algorithm to estimate large phylogenies by maximum likelihood Syst Biol 2003 52 5 696 704 10.1080/10635150390235520 14530136

[B46] Yang ZH PAML: a program package for phylogenetic analysis by maximum likelihood Comput Appl Biosci 1997 13 5 555 556 9367129 10.1093/bioinformatics/13.5.555

[B47] Hildebrand A Remmert M Biegert A Söding J Fast and accurate automatic structure prediction with HHpred Proteins 2009 77 128 132 10.1002/prot.22499 19626712

[B48] Williams-Carrier R Kroeger T Barkan A Sequence-specific binding of a chloroplast pentatricopeptide repeat protein to its native group II intron ligand RNA 2008 14 9 1930 1941 10.1261/rna.1077708 18669444 PMC2525963

[B49] Roy A Kucukural A Zhang Y I-TASSER: a unified platform for automated protein structure and function prediction Nat Protoc 2010 5 4 725 738 10.1038/nprot.2010.5 20360767 PMC2849174

[B50] Tamura K Peterson D Peterson N Stecher G Nei M Kumar S MEGA5: Molecular Evolutionary Genetics Analysis Using Maximum Likelihood, Evolutionary Distance, and Maximum Parsimony Methods Mol Biol Evol 2011 28 10 2731 2739 10.1093/molbev/msr121 21546353 PMC3203626

[B51] Anisimova M Gascuel O Approximate likelihood-ratio test for branches: A fast, accurate, and powerful alternative Systematic biology 2006 55 4 539 552 10.1080/10635150600755453 16785212

