# Supplementary figures and images for: Molecular evolution of pentatricopeptide repeat genes reveals truncation in species lacking an editing target and structural domains under distinct selective pressures
Source: BMC Evol Biol. 2012 May 14;12:66. doi: 10.1186/1471-2148-12-66 (PMC3441922; doi:10.1186/1471-2148-12-66)

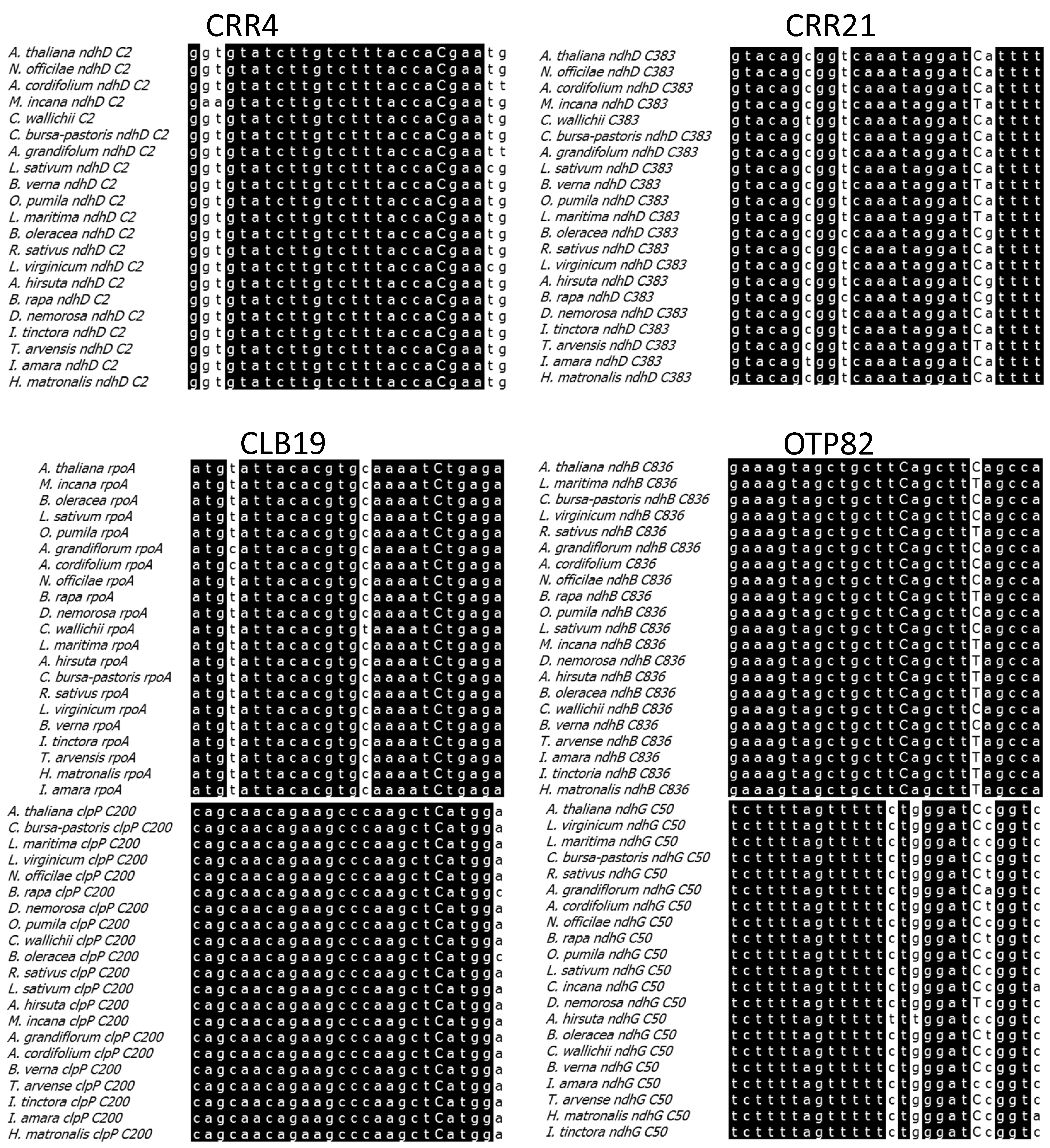

Supplement: Additional file 2 — Alignment ofcis-elements for CRR4, CRR21, CLB19, and OTP82. A figure illustrates nucleotide sequences around editing sites targeted by four PPR proteins. Each sequence represents 20 nucleotides upstream and 5 nucleotides downstream of the editing site. The editing sites are indicated capitalized characters. Nucleotides that are 100% conserved are blocked in black. [file 1471-2148-12-66-S2.tiff]

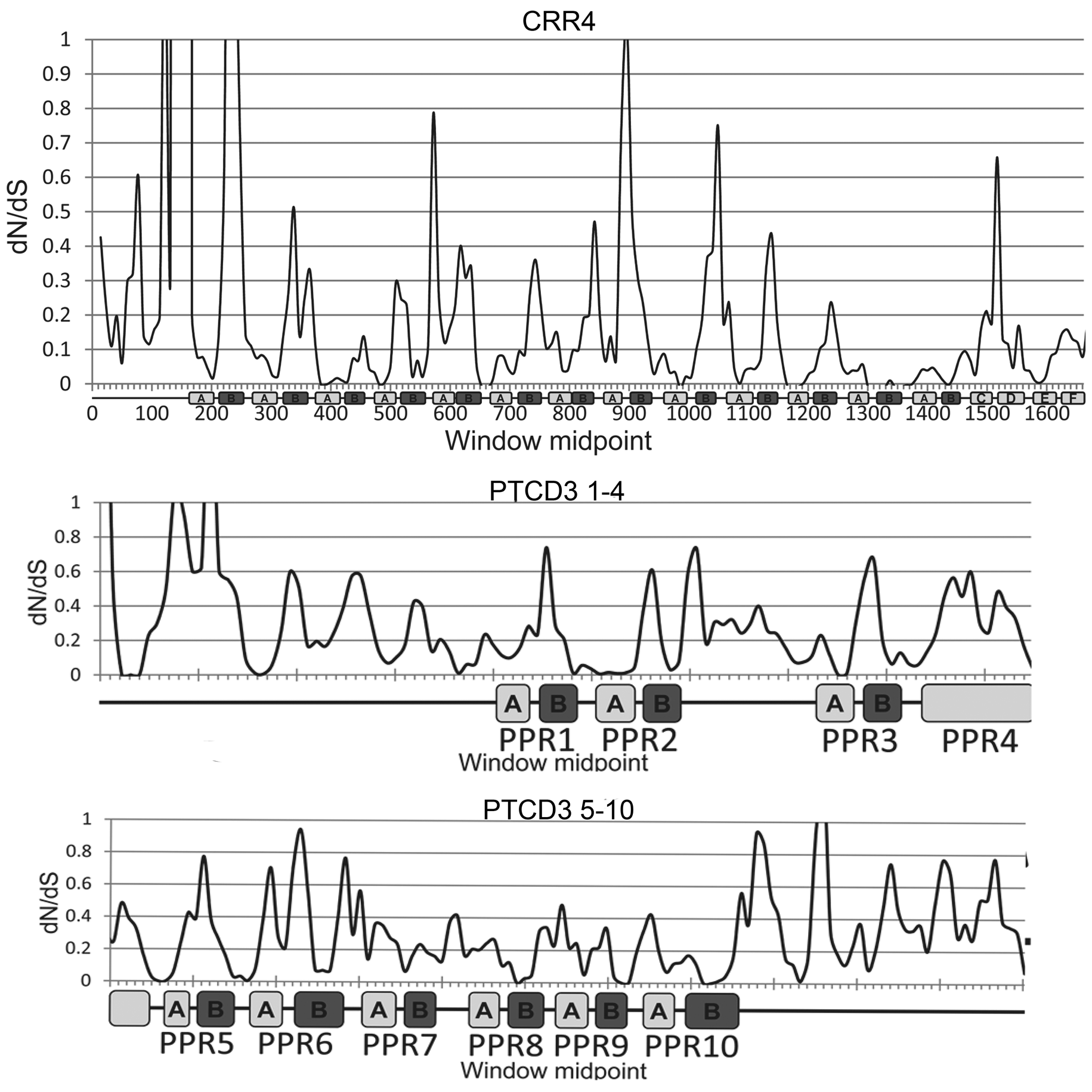

Supplement: Additional file 5 — Rate of evolution of theCRR4 genes in Brassicaceae and PTCD3 in mammals. The dN/dS values for a 27nt window are plotted versus the midpoint position of each window for CRR4 (at top) and PTCD3 (at bottom). Below the nucleotide positions the respective positions of predicted helices are indicated by labeled boxes. [file 1471-2148-12-66-S5.tiff]
