# Supplementary material for: Molecular evolution of pentatricopeptide repeat genes reveals truncation in species lacking an editing target and structural domains under distinct selective pressures
Source: BMC Evol Biol. 2012 May 14;12:66. doi: 10.1186/1471-2148-12-66 (PMC3441922; doi:10.1186/1471-2148-12-66)
Supplement: Additional file 4 — Examination of species with truncatedCRR21genes for potential duplicate copies ofCRR21. [file 1471-2148-12-66-S4.doc]

Additional File 4

The possibility of *CRR21* gene redundancy, and the potential existence of both truncated and full length orthologs, was examined in species with truncated *CRR21* genes. Initially, internal gene fragments were obtained by PCR with primers designed from highly conserved sequences within the Brassicaceae. Since these PCR products lack sequences for the important C-terminal domains, we utilized 3’ RACE to obtain the 3’ portion of *CRR21* transcripts. Based on sequences from the genomic PCR and 3’ RACE, we were able to construct sequences for *CRR21* genes and the 3’ UTR that demonstrate truncation relative to the Arabidopsis gene.

In the case of species with truncated *CRR21* genes (*Barbarea verna*, *Lobularia maritima,* and *Thlaspi arvense*), the truncated forms of the gene represent the only intact orthologs that could be amplified from genomic DNA in these species. We were unable to amplify full length products from genomic DNA with primers based on the full length Arabidopsis gene. If full length orthologs are present, the gene sequence must be sufficiently diverged to escape attempts at PCR amplification with multiple distinct primers for both forward and reverse orientations.

In order to address possible gene duplication with available genome resources, we examined *CRR21* gene architecture in a broader pool of species with sequenced genomes. Three species in the order Malpighiales have truncated CRR21 orthologs and have lost the requirement to edit the CRR21 target (*M. esculenta*, *R. communis*, and *P. trichocarpa*)*.* No full length CRR21 ortholog could be identified in the sequenced genome of any of these three species.
